# List of Reviewers 2024

**DOI:** 10.1192/bjo.2025.3

**Published:** 2025-02-12

On behalf of the editorial board, the Editor-in-Chief would like to thank the following for their contribution as peer reviewers in the period from January 2024 – December 2024. Their anonymous work for the journal forms the foundation to its success and is highly appreciated.

Mari Aaltonen

Melanie Abas

Alaa Abdalla

Elia Abi-Jaoude

Abdullahi Aborode

Danielle Adams

Olufemi Adebajo

Frances Adiukwu

Andrea Aguglia

Mike Akroyd

Hassen Al-Amin

Zainab Al-Attar

Steven Albert

George Alevizopoulos

Laith Alexander

Regi Alexander

Farhan Ali

Jon Allard

Hasanen Al-Taiar

Margarida Alves

Ayomipo Amiola

Dimitra Anastasiadou

Viola Antao

Paul Appelbaum

Alan Apter

Charlotte Archer

Urska Arnautovska

Samuel R. C. Arnold

Bill Arroyo

Muqaddas Asif

Tony Attwood

Tara Austin

Erland Axelsson

Agnes Ayton

Matilda Azis

Gloria Bachmann

Line Bager

Elena Ballante

Jordan Bamford

Yoram Barak

Skye Barbic

Bijon Baroi

Lamin FM Barrow

Christopher Bartley

Francesco Bartoli

Philip Batterham

Howard Bauchner

Joy Noel Baumgartner

Adam Bayes

Ben Beaglehole

Klaus Martin Beckmann

Massimiliano Beghi

Caroline Bell

Sarah Bendall

Domenico De Berardis

Maximus Berger

Yoav Bergman

Tomi Bergström

Suze Berkhout

Felix-Antoine Berube

Frank Besag

Tesfalidet Beyene

Vishal Bhavsar

Kamaldeep Bhui

Lucy Biddle

Catherine Bird

Mary Bitta

Marc Blom

Joseph Boden

Lana Bojanić

Marie Bolison

Linda Booij

Rohan Borschmann

Mark Bosmans

Michel Botbol

Richard Braithwaite

Serge Brand

Christian Brandt

Johanna Breilmann

Norella Broderick

Guy Brookes

Lisa Brophy

Silvia Bruzzone

Paola Bucci

Sue Buckley

Claudia Bull

Eilish Burke

Tom Burns

Hollie Burton

Michael Bushey

Rory Byrne

Camilla Cadorin

Marilia Calcia

Alison Calear

M Belén Calvo

Cemre Candemir

Adelita Cantu

Franco Cardone

Andres Camilo Cardozo Alarcon

Mauro Giovanni Carta

Virginia Carter Leno

Livia Carvalho

Patricia Casey

Francisco Castellanos

Samuel Chamberlain

Christian Chan

Sze Chi Chan

Russell Chander

Laura Chandler

Shu-Sen Chang

Wan-Chen Chang

Wing Chung Chang

Colleen Cheek

Andrew Cheng

Verity Chester

Keng-hong Chhoa

Tsai-Chin Cho

Soumya Choudhary

Michael Clark

Helgi Clayton McClure

Seonaid Cleare

Catherine Clelland

Stephen Clift

Derek Clougher

Melissa Co

Edwina Coghlan

Talia R. Cohen

Dan Cohen

Alessandro Colasanti

Pamela Collins

John Cookson

Emily Corner

Patrick Corrigan

Samuele Cortese

Jose Eduardo Cosentino

Sarah Cote

Lesley Cousins

Liz Coventry

John Crichton

Julia Crilly

Ioana Cristea

Matthew Croucher

Jacob Crouse

Alfredo Cuéllar Barboza

Alexis Cullen

Kathryn R. Cullen

Henry Cutler

Rachael Cvejic

Jan Cwik

Amber Dadwal

Frances Dark

Jayati Das-Munshi

Michael Daubney

Melissa-Claire Daugelat

Conor Davidson

Alice Davis

Edward Day

Giovanni de Girolamo

Elena De Rossi

Claire De Souza

Bertrand de Toffol

Shaystah Dean

Serhiy Dekhtyar

Tyco Dekkers

Alice Demesmaeker

Saskia Denecke

Pushpal Desarkar

Rashi Dhensa-Kahlon

Sukriti Dhingra

Jeremy Dixon

Sandra Dixon

Sabina Dizdarevic

Bettina K. Doering

Anne M. Doherty

Valeria Donisi

Nicholas Donnelly

Barbara Dooley

Katie Douglas

James Downs

John Drury

Priyanka D’Souza

Jubao Duan

Michael Dudley

Anne Duffy

Charlie Duncan

Adisti Dwijayanti

Grete Dyb

Jessica Eccles

Maria Efremova

Anna Ehrlich

Claire Enthoven

Whiskey Eromona

Javier Escobar

Elinor Eskilsson Strålin

Fernando Espi Forcen

Ben Etheridge

Chris Evans

Michael Ewbank

Chiara Fabbri

Nichole Fairbrother

Kathleen Falster

Luis Farhat

Lorna Farquharson

Nada Fath

Marc Feger

Emilio Fernandez-Egea

Augusto Ferraris

Anne Ferrey

Jane Fisher

Christopher Fitch

Tony Foley

Ted Fong

David Forbes

Tamsin Ford

Tomáš Formánek

Sarah Fortune

James Foulds

David Fraguas

Sophia Frangou

Andres Fresno

Michael Fritz

Rachael Frost

Haojie Fu

Junya Fujino

Frederick Furniss

Rafael Gafoor

Megan Galbally

Silvana Galderisi

Satheesh Kumar Gangadharan

Symielle A. Gaston

Raluca Georgescu

Ioannis Georgiou

Neera Ghaziuddin

Domenico Giacco

Katrin Giel

Kiranpreet Gill

David Gillanders

Rolf Gjestad

Shih Ee Goh

Rachel Goldenberg

Benjamin Goldstein

Leticia Gonzalez Blanco

Sarah Goodday

Joshua Gooley

Dipesh Gopal

Sarah Gordon

Doron Gothelf

Rossetos Gournellis

Nayla Grace

Roland Grad

Sandeep Grover

Matheus Guerra

Nihit Gupta

Gareth Hagger-Johnson

Jessica Hall

Samuel Hanu

Amy Hardy

David Harley

Yvonne Hartnett

Kenji Hashimoto

Aaron Hauptman

Laura Havers

Philip Hazell

Jean-Baptiste Hazo

Yun-Feng He

Shantala Hegde

Stefanie Maria Helmer

Timea Helter

Claire Henderson

Morten Hesse

Sarah Hetrick

Eileen Heumann

Michelle Heward

Andrew Hill

Andreas Hoell

Robert Hoerr

Dawn Holmes

Mariko Hosozawa

Matthew Hotopf

Louise Howard

Richard Howard

Patricia Howlin

Rebekah Huber

Vilgot Huhn

Rachael Hunter

M Omair Husain

Muhammad Husain

Le Duc Huy

Christophe Huynh

Karim Ibrahim

Dragos Iliescu

Krista Ingram

Konstantinos Ioannidis

Maria Irigoyen-Otiñano

Kimberley Iyemere

Helen Jacks

Matthew Janicki

Staffan Janson

Matthew Jay

Seyede Zohreh Jazaeri

Rachel Jenkins

Hyoseok Jeong

Zhouxin Jia

Jingqi Jiang

Katherine Johnson

Brett Jones

Michelle Jongenelis

Jenny Jordan

Anthony F. Jorm

Dan Joyce

Sun Jae Jung

Hans Jürgen Möller

Mario Juruena

Ashraf Kagee

Michail Kalfas

Lucie Kalisova

Hanna Kampling

Richard Kanaan

Soumen Karmakar

Cornelius Katona

Kenneth Kaufman

F. Kauye

Johanna Keeler

Brendan Kelly

Kimberley Kendall

Rachel Kent

Patrick Keown

André Kerber

Ronald Kessler

Bess Kew

Reza Kiani

Daeho Kim

Jochen Kindler

David Kingdon

Megan Kirk Chang

Olivia Kirtley

Steve Kisely

Hans Knoblauch

Kairi Kolves

Mary-Helen Kosmidis

Changgui Kou

Ivan Koychev

Lukas Krone

Rajesh Kumar

Veena Kumari

Satyajit Kundu

Chian-Jue Kuo

Terhi Kurko

Marinos Kyriakopoulos

Rebecca Lacey

Emmeline Lagunes-Cordoba

Matthew Large

Axel Laurell

Michail Lavdas

Anna Lavis

Stephen Lawrie

Carlo Lazzaro

Sarah Ledden

Charles Tzu Chi Lee

Yao-Tung Lee

Jeannette Lely

Henna Lemetyinen

Belinda Lennox

Timothy Leow

Peter Lepping

Jessica Letch

Jialiang Li

Jing Li

Su-Xia Li

Wenfei Li

Peter Liddle

Chung-Ying Lin

Gen-Min Lin

Hsiang-Yuan Lin

Jr-Rung Lin

Kathleen Lin

Alexander Lisinski

Jialiang Liu

Baopeng Liu

Chao Liu

Feng Liu

Tieqiao Liu

Tong Liu

Lisa Lloyd

Maria Loades

R. Loewy

Carlo Longhitano

Anikó Lovik

Hanna Lu

Peter Lucassen

James Luccarelli

Mario Luciano

Nic Ludin

Lingz Luo

Qinghua Luo

Frances Lynch

Bei Lyu

Gengyu Lyu

Jun Lyu

Naoise Mac Giollabhui

Orla Macdonald

Lucy Maconick

Irene Malaty

Mohammed A Mamun

Kenneth Man

Pedro Manfro

John Mann

Jenni Manuel

Sarah Markham

Charles Marshall

Peter Martin

Mohammad Maruf

Sergi Mas

Sebastiano Massaro

Richard Mattison

Barbara Maughan

Sebastian Max

Soraya Mayet

Michelle McCarthy

Shawn McClintock

Justin McDaniel

Brett McDermott

Alexander McFarlane

Virginia McIntosh

Theresa McIver

Neil McKenzie

Sally McManus

Fiona McNicholas

Nick Meader

Graham Mellsop

Tracy Melzer

Hannah Pasha Memon

Marcelo Mendonça

Paolo Meneguzzo

Carrington C. Merritt

Adrian Meule

Thomas Meyer

Lynden Miles

Sayantanava Mitra

Josephine Mollon

Nathan Monk

Silvia Daniela Morbelli

Christo El Morr

Markus Muehlhan

Christoph Mueller

Marie Mueller

Monika Mueller

Chen Mu-Hong

Dahlia Mukherjee

Marco Mula

Lina Münker

Roger Muñoz-Navarro

Glynis Murphy

Julian Mutz

Shadreck Mwale

Zeina Nasser

Urs Nater

Prathiba Natesan Batley

Diana Nechita

Charles B. Nemeroff

Tamsin Newlove-Delgado

Richard Newton

Qin Xiang Ng

Minh-Hoang Nguyen

Marie Nicolini

Olav Nielssen

Solja Niemelä

Joanne Noblett

Roghieh Nooripour

Emilie Nordby

Alicia Norman

Rupinder Oberoi

Aileen O’Brien

John O’Brien

Taichi Ochi

Maja O’Connor

Brian O’Donoghue

David O’Donovan

Michael O’Donovan

David Okai

Paolo Olgiati

Dominic Oliver

Kristine Olson

Linnet Ongeri

Holly O’Rourke

Dennis Ougrin

Gareth Owen

Busra Ozen-Dursun

Jon Painter

Christina Palantza

Lawrence Palinkas

Milagros Pallavicini

Christopher Palmer

Peter Panayi

Amanda Pannu

Anna Panzeri

Zacharias Papadakis

Chrysovalantis Papathanasiou

Christian Paret

Carmine Pariante

Minakshi Parikh

Joel Paris

Pornjira Pariwatcharakul

Timo Partonen

Nasir Pasha

Evgenii Pashnin

Gabriela Pavarini

Leanne Payne

Jane E. Persons

Eva Petkova

Theodore Petti

Elizabeth Pienkos

Baptiste Pignon

Grace Pike

A Pimplaskar

Alexandra Pitman

Oleguer Plana-Ripoll

Vince Polito

Rob Poole

Richard Porter

Alex Porthukaran

Stephen Potts

Albert Powers

Holly Prigerson

Jesus Privado

Joan Prudic

Elmo Pulli

Shalet Punnoose

Julian Purcell

Katie Pybus

Robin Quillivic

Inti Qurashi

Andrea Raballo

Samah Rabei

Keeta Rajeevi

Rajani Raju

Kutlwano Ramaboa

Veronica Ranieri

Abdul Raoof

Charlene Rapsey

Natalie Rasgon

Lawrence Ratna

Iris Rawtaer

John Read

Nicola Reavley

Yansen Alberth Reba

Susan Rees

Lennart Reifels

Tai Ren

Charles Reynolds

Henriette Riley

Giulia Rinaldi

Whitney Ringwald

Pauline Rivart

Abbey Robbins

Ian R. H. Rockett

Almudena Rodriguez

Rubén Rodríguez-Cano

Deblina Roy

Ashok Roy

Marc-André Roy

David Russ

Howard Ryland

Marcin Rządeczka

Laura J. Sahm

Martha Sajatovic

Shinji Sakamoto

Ayman Saleh

Jay Salpekar

Zainab Samaan

Ludovic Samalin

Chase Samsel

Ignacio Sandia Saldivia

Paramala Santosh

Meghri Sarkissian

Ananya Sarma

Rob Saunders

Mark Scheepers

Philip Schluter

Ulrike Schmidt

Maude Schneider

Lisa Schölin

K Oliver Schubert

Thomas Schulze

Asilay Seker

Edward Selby

Gianluca Serafini

Alexandre Serpa

Maayan Shacham

Shumaila Shahbaz

Aparna Shaji

Pak Chung Sham

Diana Shamsutdinova

Fiona Shand

Rohit Shankar

Ivan Shanley

Stav Shapira

Omer Shareef

Sifat Sharmin

Ben Shaw

Alison Shea

Andrew Shepherd

Ashley Brianna Sheppard

Ruth Shim

Paul Shotbolt

Gemma Sicouri

Justyna Sierpatowska

Cristiane Silvestre de Paula

Alan Simpson

Ajit Singh

Sukhmeet Singh

Swaran Singh

Marcin Siwek

Karen Slade

Abigail Smakowski

Gregory Smith

Lade Smith

John Snowdon

John Söderholm

Luke Solomons

Dahee Son

Menghuan Song

Kimmo Sorjonen

Helene Speyer

Kousik Surya Sridharan

Anamika Srivastava

Pauline Stas

Jill Stavert

Thomas Steare

Tilman Steinert

Anne Stewart

Robert Stewart

Julia Stingl

Hanni Stoklosa

Rebecca Strawbridge

Simon Stuart

Ruqayya Sulaiman-Hill

Anne-Laure Sutter-Dallay

Takefumi Suzuki

Anne Sved Williams

Cynthia Swenson

Peter Szatmari

Dionysios Tafiadis

Lut Tamam

Rajesh R Tampi

Eric Tan

Jacinta Tan

Jie Tang

Sandila Tanveer

Leila Tarokh

Pamela Taylor

Kevin Teoh

Anupam Thakur

James Thompson

Alex Thomson

Lindsay Thomson

Nicollette Thornton

Eren Timurtaş

Victoria Tischler

Louise Tofts

Samuel Tomczyk

John Torous

Luis Torres

Marie Tournier

Samuel Tromans

Mike Trott

Shih-Jen Tsai

Phillip Tully

Shmuel Tyano

Freya Tyrer

Peter Tyrer

Nian-Sheng Tzeng

Stacy Tzoumakis

Riet Van Bork

Jaap van der Stel

Roos van Westrhenen

David E. Vance

Lakshmi Venkatraman

Pieter Ventevogel

Maj Vinberg

Jasper Vöckel

Birgit Vollm

Gentian Vyshka

Juliet Wakefield

Fredrik Walby

Susan Walker

Sebastian Walther

Jianbing Wang

Meng-Ting Wang

Qianjin Wang

Shibin Wang

Taiyao Wang

Philip Ward

Nicola Warren

Judith Watson

Deborah Wearne

Matthew Webb

Imogen Wells

Mindy Westlund Schreiner

Margaret Westwater

Markus Wettstein

Rob Whitley

Sarah Whittle

Adam Wichniak

Felix Wicke

Kay Wilhelm

Simon Wilksch

Richard Williams

Claire Wilson

Jack Wilson

Victoria Wing

Emma Wolverson

Alan Wong

Irene Yuen Fung Wong

Roger Wong

Amanda Woodrow

Miranda Worthen

Ben Wright

Kim Wright

Kirsten Wright

Jingwei Wu

Kai Wu

Jukrit Wungrath

Til Wykes

Danielle Wyman

Hao Yan

Min Yang

Gregory Yates

Alua Yeskendir

Jiahui Yin

Kishwen Kanna Yoga Ratnam

Dong Keon Yon

Allan Young

Michael Young

Denis Yukhnenko

Wan Mohd Yunus

Orestis Zavlis

Michelle Zechner

Stephanie Zellers

Nanhua Zhang

Yunshu Zhang

Jonathan Zipursky

Yaara Zisman-Ilani

